# Key factors associated with oral health-related quality of life in Sri Lankan adolescents: a cross sectional study

**DOI:** 10.1186/s12903-021-01569-1

**Published:** 2021-04-29

**Authors:** Uttara Amilani, Prasanna Jayasekara, Hannah E. Carter, Sameera Senanayake, Sanjeewa Kularatna

**Affiliations:** 1 Ministry of Health, No. 385, Rev. Baddegama Wimalawansa Thero Mawatha, Colombo 10, Sri Lanka; 2 Austrailan Centre for Health Services Innovation, School of Public Health and Social Work, Queensland University of Technology, Brisbane, QLD Australia

**Keywords:** Dental diseases, Oral health-related quality of life, OIDP, Effects, Associations, Youth, Adolescents, Sri Lanka

## Abstract

**Background:**

Oral Health Related Quality of Life (OHRQoL) measures have emerged as an important oral health outcome that is able to reveal the subjective burden of illness due to oral diseases. The association between sociodemographic and socioeconomic factors, clinical dental conditions and OHRQoL indicators has been investigated in adolescent populations across the world. The purpose of this study was to investigate key factors associated with oral health-related quality of life of Sri Lankan adolescents.

**Methods:**

A cross sectional study was conducted in a sample of 15–19 year-old secondary school students in the Gampaha district of Sri Lanka. The data was collected using two self-administered questionnaires. A modified Sinhalese version of the Oral Impact on Daily Performance (OIDP) questionnaire that has been validated for Sri Lankan adolescents was administered. A second questionnaire collected information on socioeconomic characteristics, oral health care seeking and oral health behaviours. A clinical oral examination was performed on each participant. Oral health related quality of life was measured using OIDP domains and total OIDP scores. Poisson regression was used to investigate the key factors associated with the OIDP additive score.

**Results:**

A total of 1332 adolescents participated in the study. Negative quality of life impacts were more prevalent in the social and psychological domains of OIDP as compared with the functional domain. Total OIDP scores ranged from 0 to 36 with a mean of 3.16 (SD = 4.71). The multivariable analysis revealed that increasing age, low income, brushing teeth only once per day, and increased number of decayed teeth were found to be associated with poor overall OHRQoL, while male gender, frequent oral healthcare seeking patterns and absent dento-facial anomalies were associated with good OHRQoL.

**Conclusion:**

This study identified modifiable behavioural and oral health related factors which are associated with OHRQoL in Sri Lankan adolescents. Oral health interventions should target these modifiable factors to improve the OHRQoL in these populations.

**Supplementary Information:**

The online version contains supplementary material available at 10.1186/s12903-021-01569-1.

## Introduction

Clinical parameters measuring oral health are usually objective, requiring a dental professional’s judgment. Although these clinical measures indicate the presence and severity of an oral condition, they have limited utility in assessing the functional and psychological aspects of oral health in an individual [1–4]. Oral Health Related Quality of Life (OHRQoL) measures have emerged as an important oral health outcome that is able to reveal the subjective burden of illness due to oral diseases. Such measures may also be used to identify priority groups for public health interventions, and as outcome measures for oral health promotion activities [5].

A number of questionnaires are available to measure socio dental indicators in a population, including the Oral Impact on Daily Performance (OIDP) tool [6]. The OIDP is conceptually based on the International Classification of Impairments, Disabilities and Handicaps created by WHO in 1980 [7], and validated on a group of Sri Lankan Adolescents [8]. The association between sociodemographic and socioeconomic factors, clinical dental conditions and OHRQoL indicators has been investigated in adolescent populations across the world. These studies have revealed that poor OHRQoL in children and adolescents was associated with unfavourable socioeconomic conditions and poor oral health [9, 10].

A recently published study on Brazilian adolescents aged 15–19 years found that income inequality during childhood was associated with poor OHRQoL [11]. In addition, OHRQoL was negatively associated with poor individual socioeconomic indicators, high number of untreated tooth decay and missing teeth, and poor gingival status [12]. Although dental caries were statistically significantly associated with children’s OHRQoL in this study, other studies have reported non-significant findings [1, 4, 13, 14]. However, methodological limitations in sampling, data collection and analysis were acknowledged in some of these previous studies [15–17].

The purpose of this study was to investigate key factors associated with oral health-related quality of life (OHRQoL) in a representative sample of adolescents, Gampaha district, Sri Lanka. The null hypothesis was that none of the tested factors were associated with OHRQoL.

## Methods

### Study setting, sample size and study design

We report on a cross sectional study conducted in the Gampaha district in the Western Province of Sri Lanka. The study population was 15–19 year old adolescents who were attending secondary government schools in the district, encompassing a total of 377 functioning schools. The sample size for the study was calculated using the formula n = z^2^ p (1 − p)/ d^2^ [18] which produced a minimum sample of 384. Since the study was designed to adopt a cluster sampling technique, the sample size was adjusted to include the anticipated effect size of 2.9 [19]. Hence the final sample size was calculated as 1337 allowing for a 20% non-response rate. As this study involved secondary school children (above Grade 6) the minimum number of children per cluster was set to 20, reflecting a typical classroom size. A total of 67 clusters were selected using a multi stage cluster sampling technique with probability proportionate to size of grade, across grades 10, 11, 12 and 13. Clusters were selected according to the school sampling frame.

This study received approval from the Ethics Review Committee of the Faculty of Medicine, University of Colombo, Sri Lanka (Ref. No. EC 15-171). The field team was formed by a dental surgeon who carried out an oral examination on all participants; a retired school dental therapist for recording clinical data; and an assistant. Adolescents with dental problems were referred to the nearest government dental clinic where treatment was guaranteed.

### Oral impact on daily performance scale (OIDP scale)

The OIDP index was initially developed by Adulyanon [6] and later modified and validated in a sample of 220 Sri Lankan adolescents aged 15–19 [8]. During cross-cultural adaptation of the questionnaire, some items were modified and the scoring system was revised to report only the severity of the impact with a recall period of three months.

Concurrent validity of the modified OIDP was assessed by testing the scale against self-reported perceived oral treatment need and perceived oral health problems after the factor analysis. The relationships were significant (*p* < 0.05) indicating that the instrument could adequately discriminate between adolescents who did and did not have perceived dental treatment needs, and adolescents who had different perceptions of overall health problems.

The modified OIDP questionnaire contains eight items across two domains: functional; and, social and psychological. The functional domain includes items assessing the impact of oral health on: chewing and enjoying foods; talking and pronouncing clearly; and cleaning teeth. The social and psychological domain includes items assessing the impact of oral health on: good sleep without disturbances; being able to smile without embarrassment; maintaining usual emotional state without being irritable; school and household activities; and enjoying time with friends. Further detail on the modified OIDP is included in Additional files 1, 2, 3, 4 and 5.

Revised OIDP scores were recorded on a six-point likert scale to reflect how severe the impact of each event was over the past three months, ranging from 0 (indicating no impact) to 5 (indicating a very severe impact). The total OIDP scores for individual domains were calculated as the sum of the response code. The potential functional domain and social/psychological domain scores ranged from 0–15 to 0–25 respectively. Total OIDP scores could range from 0 to 40. Higher OIDP scores indicated poorer OHRQoL. The primary outcome applied in this study was the total OIDP score. Domain specific scores were analysed as secondary outcomes.

### Socioeconomic characteristics, oral health care seeking and oral health behaviour questionnaire

An additional questionnaire was developed to collect information about adolescents’ age, gender, family income (measured in Sri Lankan rupees and categorized in tertiles), oral hygiene habits (brushing frequency), consumption patterns of soft drinks, sugary items and fruits (categorized based on the frequency of consumption: occasional or regular); oral care seeking pattern (frequency of seeking care categorized according to the number of visits per year).

### Clinical examination

The children were examined in a classroom at the school lying on an ordinary desk under natural light. The clinical examination was exclusively visual, with the help of a dental mirror, CPI probe and a millimetre ruler. Biosafety measures established by the World Health Organization (WHO) were strictly followed [20]. The WHO criteria for the diagnosis of Decayed, Missing and Filled teeth (DMFT) were applied. The DMFT was categorized into caries free (DMFT = 0), low severity (DMFT = 1–4) and high severity (DMFT > 4). Oral hygiene was assessed by Oral Hygiene Index-Simplified (OHI-S). Debris and calculus indices were calculated by OHI-S using the standard formula. OHI-S scores were categorized into good oral hygiene (OHI-S = 0) and poor oral hygiene (OHI-S > 0). Periodontal status was measured by assessing the bleeding status and pocket depth. Dental trauma data were analysed according to the presence of at least one kind of trauma, or the absence of trauma. Dento-facial anomaly data were classified according to the need for professional intervention and the criteria were: 0 = none; 1 = slight but no treatment needed; 2 = severe anomalies needing treatment.

### Quality control

Quality control measures included a discussion of all possible classifications and criteria used in the study for the diagnosis of each oral health condition through an analysis of pictures of clinical cases for the disorders and diseases. An instruction manual for the field team was prepared and used during the training and throughout the data collection. Preceding the study, inter-examiner agreements were established. A specialist in community dentistry at Dental Institute, Colombo was considered the gold standard and training involved 20 children of the same age outside the study sample to test methodology. The inter examiner reliability was assessed using Kappa statistics. It showed a perfect agreement for both dental caries and bleeding. There was an 85.7% agreement for dental caries and 88.3% agreement for bleeding.

### Data analysis

Statistical analyses were performed using the Statistical Package for Social Sciences (version 23). Total OIDP scores, the overall mean OIDP score, and scores for the individual domains were analysed for differences between specific oral diseases and disorders, and adjusted for socio economic characteristics and oral health behaviours. After applying statistical and graphical tests for normality, it was observed that the distribution was positively skewed; hence non-parametric tests were used predominantly. Mann Whitney tests were used to compare the OIDP scores between different levels of dental caries, oral hygiene, trauma and malocclusion, with the level of significance was set to 5% (*p* < 0.05). Spearman correlation was used to assess the correlation between the OIDP scores with DMFT, OHI-S, debris and calculus indices.

Poisson regression models were used to determine factors associated with the total OIDP score, functional domain score and social and psychological domain score. The independent variables in these regressions included sociodemographic characteristics (adolescents’ age, gender, family income and mothers’ education level), oral hygiene habits (daily brushing frequency), consumption patterns of soft drinks, sugary items and fruits and oral disease conditions (presence of dental trauma, anomaly, bleeding and pocketing and as well as number of decayed, filled and missing teeth due to caries and OHI-S index). The study conformed to the STROBE guidelines.

## Results

A total of 1,340 adolescents were approached to participate in the study. The final sample was 1,332 giving a participation rate of 99% (Table 1). All adolescents completed the questionnaire independently. No questions were excluded from the data analysis due to incompleteness of data. The mean age of the children was 16.5 years (SD = 1.25) and approximately 47% were boys. Maternal education varied from elementary level of schooling to university education, with 62.7% of mothers having a highest education level of elementary or middle level schooling. Income was relatively low with approximately 50% of families in the lowest income tertile. Dental caries were present in 47.6% of the sample and the mean DMFT was 1.14 (SD = 1.63).

**Table 1:** Socio demographic and clinical characteristics of the sample of children (n = 1332)

Variables	N (%)
Gender	
Male	623 (46.8)
Female	709 (53.2)
Mother’s education	
Elementary/middle school (up to O/L)	835 (62.7)
High school (up to graduate)	497 (37.3)
Family income	
1st tertile (≥ Rs. 25,000.00)	662 (49.7)
2nd tertile (< Rs. 25,000.00 to > 50,000.00)	494 (37.1)
3rd tertile (≤ Rs. 50,000.00)	176 (13.2)
Oral health care seeking pattern	
Infrequent (never)	378 (28.4)
Frequent (more than 1 visit per year)	954 (71.6)
Frequency of daily tooth brushing	
Once	358 (26.9)
Twice or more	974 (73.1)
Consumption pattern of sugary items (candies/ sugary snacks)	
Occasional	394 (29.6)
Regular	938 (70.4)
Consumption pattern of soft drinks	
Occasional	853 (64)
Regular	479 (36)
Consumption pattern of fruits	
Occasional	653 (49)
Regular	679 (51)
Dental caries experience (DMFT)	
Caries free (DMFT = 0)	697 (52.3)
Low severity (DMFT ≥ 1 to ≤ 4)	568 (42.6)
High severity (DMFT ≥ 5)	67 (5)
Oral hygiene (OHI-S)	
Good (OHI-S = 0)	589 (44.2)
Poor (OHI-S ≥ 1)	743 (55.8)
Bleeding gums	
Absent	1073 (80.6)
Present	259 (19.6)
Tooth pocketing	
Absent	1310 (98.3)
Present	22 (1.7)
Dental trauma	
Absent	1305 (98)
Present	27 (2)
Dento facial anomaly	
Absent	1067 (80.1)
Present	265 (19.9)

Table 2 displays the distribution of the responses to the OIDP according to each question. Negative impacts were more prevalent in the social & psychological domain relative to the functional domain. Items relating to enjoying time with friends, maintaining usual emotional state without being irritable and being able to smile without embarrassment were the most frequently reported impacts on the social & psychological domain. Chewing and enjoying foods was the most frequently reported impact on the functional domain.

**Table 2:** Oral impact on daily performance scale responses (n = 1332)

Impact	No / very little/little impact	Average impact	Severe/very severe impact
	N (%)	N (%)	N (%)
Social and psychological domain			
Impact on good sleep without disturbances	1300 (97.6)	20 (1.5)	12 (0.9)
Impact on being able to smile without embarrassment	1241 (93.2)	53 (4)	38 (2.9)
Impacts on maintaining usual emotional state without being irritable	1238 (92.9)	56 (4.2)	38 (2.9)
Impact on school and household activities	1306 (98)	16 (1.2)	10 (0.8)
Impact on enjoying with friends	1166 (87.5)	114 (8.6)	52 (3.9)
Functional domain			
Impact on chewing and enjoying foods	1276 (95.8)	50 (3.8)	6 (0.5)
Impact on talking and pronouncing clearly	1299 (97.5)	22 (1.7)	11 (0.8)
Impact on cleaning teeth	1304 (97.9)	185 (1.4)	10 (0.8)

The OIDP scores ranged from 0 to 36 with a mean of 3.16 (SD = 4.71). When mean overall score was analysed, it was evident that health care seeking pattern, consumption pattern of sugary items and soft drinks, presence of dental trauma and presence of dento-facial anomaly had a statistically significant negative impact on OHRQoL (Table 3).

**Table 3:** Mean differences between selected oral clinical conditions and characteristics of oral behaviours for each domain and for overall OIDP (n = 1332)

	Functional domain	Social and psychological domain	Total OIDP
	Mean (SD)	Mean (SD)	Mean (SD)
Overall	0.87 (1.72)	2.29 (3.40)	3.16 (4.71)
Oral health care seeking pattern			
Infrequent	0.68 (1.42)	1.95 (3.18)	2.63 (4.18)
Frequent (1 visit per year)	0.94 (1.82)	2.43 (3.48)	3.37 (4.90)
*P* value^a^	0.011^a^	< 0.001^a^	< 0.001^a^
Frequency of daily tooth brushing			
Once	1.07 (1.82)	2.55 (3.89)	3.62 (5.23)
Twice or more	0.79 (1.63)	2.19 (3.20)	2.99 (4.50)
*P* value^a^	< 0.001^a^	0.229	0.49
Consumption pattern of sugary items (candies/ sugary snacks)			
Occasional	0.73 (1.48)	1.90 (2.80)	2.62 (3.76)
Regular (daily)	0.93 (1.81)	2.46 (3.62)	3.38 (5.04)
*P* value^a^	0.066	0.006^a^	0.008^a^
Consumption pattern of soft drinks			
Occasional	0.79 (1.54)	2.00 (2.90)	2.79 (4.03)
Regular (daily)	1.00 (1.99)	2.82 (4.10)	3.82 (5.68)
*P* value^a^	0.257	0.001^a^	0.004^a^
Consumption pattern of fruits			
Occasional	0.83 (1.61)	2.30 (3.33)	3.13 (4.54)
Regular (daily)	0.91 (1.81)	2.28 (3.47)	3.19 (4.87)
*P* value^a^	0.815	0.961	0.932
Bleeding gums			
Absent	0.84 (1.70)	2.32 (3.42)	3.16 (4.68)
Present	0.98 (1.77)	2.18 (3.35)	3.16 (4.84)
*P* value^a^	0.120	0.958	0.765
Tooth pocketing			
Absent	0.86 (1.71)	2.29 (3.42)	3.16 (4.72)
Present	1.14 (1.98)	2.23 (2.67)	3.36 (4.42)
*P* value^a^	0.448	0.564	0.423
Dental trauma			
Absent	0.86 (1.72)	2.27 (3.40)	3.14 (4.71)
Present	1.07 (1.77)	3.22 (3.66)	4.30 (4.92)
*P* value^a^	0.386	0.052	0.037^a^
Dento facial anomaly			
Absent	0.77 (1.62)	2.17 (3.27)	2.95 (4.51)
Present	1.26 (2.01)	2.76 (3.88)	4.02 (5.39)
*P* value^a^	< 0.001^a^	0.006^a^	< 0.001^a^

^a^*p* < 0.05

Mann–Whitney test was used

Table 4 displays the correlations between selected oral health conditions between domain specific scores and with the total OIDP score. While the DMFT index and number of decayed teeth had significant positive correlations with both domains as well as with the total OIDP score, the strength of association was relatively small (Table 4).

**Table 4:** Correlation between selected oral clinical conditions for each domain and for overall OIDP (n = 1332)

	Functional domain	Social and psychological domain	Total OIDP
	Spearman r	Spearman r	Spearman r
DMFT index	0.108^a^	0.133^a^	0.14^a^
Number of decayed teeth	0.121^a^	0.121^a^	0.13^a^
Number of filled teeth due to caries	0.041	0.066^a^	0.068^a^
Number of missing teeth due to caries	0.017	0.037	0.035
OHI-S index	0.001	0.019	0.012
Debris index (DI)	0.001	0.036	0.029
Calculus index (CI)	0.030	− 0.003	0.000

Spearman correlation test were used

^a^Correlations significant at the 0.05 level

Table 5 outlines the results of the multi-variable analyses of the association between total OIDP score, individual domain scores and exploratory variables. Increasing age, low income, brushing teeth only once per day and increased number of decayed teeth were found to be associated with poor overall OHRQoL as well as poor functional and social and psychological domains. Male gender, frequent oral healthcare seeking pattern and absent dento-facial anomaly were associated with lower scores (good OHRQoL) in total and in the individual domain scores. Absence of dental trauma was associated with higher overall OHRQoL and social and psychological related quality of life.

**Table 5:** Poisson regression between total OIDP score, functional and social and psychological domains with exploratory variables (n = 1332)

Variable	Total OIDP score			Functional domain			Social & psychological domain		
	Exp (β)	95% CI	*P*	Exp (β)	95% CI	*p*	Exp (β)	95% CI	*p*
Gender									
Female	Ref.			Ref.			Ref.		
Male	0.89	0.83–0.95	< 0.001	0.84	0.75–0.95	0.007	0.91	0.84–0.98	0.016
Age	1.12	1.09–1.15	< 0.001	1.15	1.10–1.21	< 0.001	1.10	1.07–1.14	< 0.001
Mother’s education									
Elementary/middle school (up to O/L)	Ref.			Ref.			Ref.		
High school (up to graduate)	1.13	1.06–1.21	< 0.001	1.04	0.92–1.18	0.465	1.17	1.08–1.26	< 0.001
Family income									
1st tertile (≥ Rs. 25,000.00)	1.22	1.11–1.35	< 0.001	1.22	1.00–1.47	0.041	1.23	1.09–1.38	< 0.001
2nd tertile (< Rs. 25,000.00 to > 50,000.00)	0.99	0.89–1.10	0.935	1.04	0.85–1.26	0.69	0.98	0.87–1.11	0.823
3rd tertile (≤ Rs. 50,000.00)	Ref.			Ref.			Ref.		
Oral health care seeking pattern									
Infrequent	Ref.			Ref.			Ref.		
Frequent	0.80	0.74–0.86	< 0.001	0.73	0.63–0.84	< 0.001	0.82	0.75–0.90	< 0.001
Frequency of daily tooth brushing									
Once	Ref.			Ref.			Ref.		
Twice or more	1.19	1.11–1.27	< 0.001	1.33	1.17–1.51	< 0.001	1.14	1.05–1.23	0.001
Consumption pattern of sugary items									
Occasional	Ref.			Ref.			Ref.		
Regular	1.11	1.04–1.14	< 0.001	1.18	1.10–1.21	< 0.001	1.02	1.004–1.11	< 0.001
Consumption pattern of fruits									
Occasional	Ref.			Ref.			Ref.		
Regular	0.99	0.93–1.06	0.951	0.88	0.78–1.003	0.055	1.04	0.96–1.12	0.305
Bleeding gums									
Absent	Ref.			Ref.			Ref.		
Present	1.04	0.95–1.14	0.377	0.91	0.77–1.08	0.327	1.09	0.98–1.22	0.094
Tooth pocketing									
Absent	Ref.			Ref.			Ref.		
Present	0.96	0.75- 1.22	0.755	0.91	0.60–1.39	0.681	0.98	0.73–1.33	0.936
Dental trauma									
Present	Ref.			Ref.			Ref.		
Absent	0.79	0.66–0.96	0.018	0.93	0.64–1.36	0.732	0.75	0.60–0.93	0.009
Dento facial anomaly									
Present	Ref.			Ref.			Ref.		
Absent	0.74	0.69–0.80	< 0.001	0.63	0.55–0.72	< 0.001	0.79	0.73–0.87	< 0.001
Number of decayed teeth	1.07	1.05–1.10	< 0.001	1.12	1.07–1.17	< 0.001	1.06	1.03–1.09	< 0.001
Number of missing teeth due to caries	1.07	1.004–1.15	0.039	1.15	1.01–1.31	0.032	1.05	0.96–1.14	0.247
Number of filled teeth due to caries	1.07	1.03–1.10	< 0.001	1.03	0.96–1.10	0.391	1.08	1.04–1.12	< 0.001
Debris index (DI)	0.93	0.84–1.02	0.166	0.78	0.64–0.94	0.012	1.002	0.89–1.12	0.969
Calculus index (CI)	1.09	0.97–1.22	0.143	1.27	1.02–1.58	0.033	1.02	0.89–1.17	0.752

## Discussion

This study identified seven key factors that were associated with OHRQoL in a sample of Sri Lankan adolescents. To our knowledge, this is the first study to report on domain specific OIDP scores in Sri Lankan adolescents after the recent validation of the tool in this population.

The importance of OHRQoL is particularly relevant for adolescents. There is evidence that juveniles are more sensitive to a variety of impacts, such as appearance, relative to older age groups. These impacts may affect quality of life and influence social skills and education [21, 22]. This is supported by our findings that social and psychological impacts; such as enjoying time with friends and smiling without embarrassment, were more prevalent than those observed in studies reporting on adults and elderly [23].

Our data were strongly skewed towards to the “no impact” or “very little/little impact” end of the scale, with more than 85% of the study population reporting they had not experienced an oral impact during past three months, giving a strong floor effect. This is similar to findings in previous studies among children in Brazil [15, 24]. This OIDP distribution of scores is characteristic of a population based study and indicative of adolescents having genuinely low levels of impacts, but may be due the instrument not being sensitive to identify the impacts that are experienced in the particular cultural context. Direct comparisons with the published literature across different countries must be interpreted with caution as the nature and the magnitude of impacts may vary among the populations with different cultural backgrounds [25–27]. The prevalence of oral impacts experienced during the previous three months by the study population was less than those reported in some previous studies [28, 29], with values slightly lower than those reported in other young Asian populations [27, 30, 31].

Frequency of daily tooth brushing appeared to have a significant association with the functional domain, whereas number of filled teeth, consumption of sugary items and soft drinks showed a significant association with the social and psychological domain. Oral health care seeking pattern, number of decayed teeth and presence of dento facial anomaly were significantly associated with both domains. These finding were similar to that found in published literature on Brazilian children [15, 24].

Our findings revealed that a frequent oral health care seeking pattern was a protective factor for a good OHRQoL after adjustment for confounding. This reflects dental care workers’ influence on improving oral health related quality of life. Our finding that increased frequency of daily tooth brushing was a protective factor for a good OHRQoL was consistent with similar observations that have been reported in school children in Italy and New Zealand [23, 27].

Our results indicate that increasing age, low income level, brushing teeth only once per day, regular consumption of sugary items and presence of increased number of decayed teeth were risk factors for a poor OHRQoL, after adjusting for other factors. The most significant risk factor for developing dental caries and enamel erosion is the local action of the diet on teeth. Previous studies have recommended to reduce the frequency of consumption of foods containing free sugars to four times a day and to limit the total amount of free sugars consumed [32].

Recent studies have revealed that malocclusion plays an important role in social interactions and psychological well-being in adolescents [15, 33, 34] and it has been suggested that there is a significant impact of malocclusion on the OHRQoL of young children. Our findings confirm this, with absence of dento-facial anomaly identified as a protective factor for good OHRQoL after adjustment for other factors. Our results were also consistent with previous studies that have found the presence of missing teeth had a measurable effect on OHRQoL among adolescents [24, 34]. Missing teeth, whether replaced or not, may contribute to lower levels of oral well-being [32].

A key strength of this study was the use of an OHRQoL tool that has been validated in this population. Further, our data was from a relatively large sample that can be considered representative of adolescents in Gampaha district, Sri Lanka.

A limitation of our study is its cross sectional nature. It is known that cross sectional studies may be constrained in relation to hypothesis testing since the data on risk factors and outcomes are assessed at the same time [35]. Nonetheless, are findings are broadly consistent with the published literature. The use of self-report data on socioeconomic characteristics and oral health care behaviours may have introduced response bias that we were not able to account for. The results may not be generalizable to the broader Sri Lankan population, as population characteristics and service availability varies across the country. Future studies should assess the impacts of oral diseases and socioeconomic factors on oral health-related quality of life in other districts, and ideally with longitudinal study designs.

## Conclusions

This study confirms that non modifiable factors including age and gender, and modifiable factors including frequency of brushing, oral health care seeking pattern, presence of decayed teeth, presence of dento-facial anomaly and presence of dental trauma, were significantly associated with OHRQoL in Sri Lankan adolescents.

## Supplementary Information

**Additional file 1.** Cultural adaptation of item modification of OIDP scale.

**Additional file 2.** Factor analysis of modified OIDP 8 items.

**Additional file 3.** Reliabiloity analysis-Interitem correlation for the 8 items of the modified OIDP.

**Additional file 4.** Reliability analysis-corrected item total correlation.

**Additional file 5.** Concurrent validity test for the modified OIDP scores between different categories of related outcome variables.

## Data Availability

The datasets used and/or analyses during the current study are available from the corresponding author on reasonable request.

## Abbreviations

OHRQoL: Oral Health Related Quality of Life
OIDP: Oral impact on daily performance
SD: Standard deviation
WHO: World Health Organization
DMFT: Decayed, missing and filled teeth
OHI-S: Oral hygiene index-simplified
