# Supplementary material for: Key factors associated with oral health-related quality of life in Sri Lankan adolescents: a cross sectional study
Source: BMC Oral Health. 2021 Apr 29;21:218. doi: 10.1186/s12903-021-01569-1 (PMC8082852; doi:10.1186/s12903-021-01569-1)
Supplement: Supplementary file 1 — Additional file 1. Cultural adaptation of item modification of OIDP scale. [file 12903_2021_1569_MOESM1_ESM.docx]

**Supporting material 1**

**Table 1** Cultural adaptation and item modification of OIDP scale

| **No** | **Performances assessed in original tool** | **Item included in the modified tool** |
| --- | --- | --- |
| 1 | Eating and enjoying food | Impact on chewing and enjoying foods |
| 2 | Speaking and pronouncing clearly | Impact on talking and pronouncing clearly |
| 3 | Cleaning teeth | Impact on cleaning teeth |
| 4 | Sleeping and relaxing | Impact on good sleep without disturbances |
| 5 | Smiling, laughing and showing teeth without embarrassment | Impact on being able to smile without embarrassment |
| 6 | Maintaining usual emotional state without being irritable | Impacts on maintaining usual emotional state without being irritable |
| 7 | Carrying out major work or social role | Impact on school and household activities |
| 8 | Enjoying contact with people | Impact on enjoying time with friends |
