# Supplementary material for: Key factors associated with oral health-related quality of life in Sri Lankan adolescents: a cross sectional study
Source: BMC Oral Health. 2021 Apr 29;21:218. doi: 10.1186/s12903-021-01569-1 (PMC8082852; doi:10.1186/s12903-021-01569-1)
Supplement: Supplementary file 2 — Additional file 2. Factor analysis of modified OIDP 8 items. [file 12903_2021_1569_MOESM2_ESM.docx]

Supporting material 2

**Table 2** Factor analysis of modified OIDP 8 items (n=220)

|  | **Modified OIDP items** | **Factor^a^** |  |
| --- | --- | --- | --- |
| Q | Question | Factor 1 | Factor 2 |
| Q1 | Impact on chewing and enjoying foods | **0.545** |  |
| Q2 | Impact on talking and pronouncing clearly | **0.791** |  |
| Q3 | Impact on cleaning teeth | **0.960** |  |
| Q4 | Impact on good sleep without disturbances |  | **0.672** |
| Q5 | Impact being able to smile without embarrassment | 0.462 | 0.391 |
| Q6 | Impacts on maintaining usual emotional state without being irritable |  | **0.864** |
| Q7 | Impact on school and household activities |  | **0.837** |
| Q8 | Impact on enjoying time with friends |  | **0.910** |

Results from oblimin rotation with Kaiser Normalization: Bold types indicates loading >0.5 ^a^ ;Factor 1: Functional , Factor 2: Social and Psychological
