# Supplementary material for: Key factors associated with oral health-related quality of life in Sri Lankan adolescents: a cross sectional study
Source: BMC Oral Health. 2021 Apr 29;21:218. doi: 10.1186/s12903-021-01569-1 (PMC8082852; doi:10.1186/s12903-021-01569-1)
Supplement: Supplementary file 3 — Additional file 3. Reliabiloity analysis-Interitem correlation for the 8 items of the modified OIDP. [file 12903_2021_1569_MOESM3_ESM.docx]

Supporting material 3

**Table 3** Reliability analysis: Inter-item correlation for the 8 items of the modified OIDP (n= 220)

| Performance Scores | Chewing | Talking | Cleaning | Sleeping | Smiling | Emotion | Activities | Enjoying |
| --- | --- | --- | --- | --- | --- | --- | --- | --- |
| Chewing | 1.0 |  |  |  |  |  |  |  |
| Talking | 0.50 | 1.0 |  |  |  |  |  |  |
| Cleaning | 0.43 | 0.62 | 1.0 |  |  |  |  |  |
| Sleeping | 0.51 | 0.54 | 0.39 | 1.0 |  |  |  |  |
| Smiling | 0.33 | 0.62 | 0.39 | 0.52 | 1.0 |  |  |  |
| Emotion | 0.42 | 0.43 | 0.24 | 0.60 | 0.48 | 1.0 |  |  |
| Activities | 0.44 | 0.50 | 0.36 | 0.65 | 0.52 | 0.72 | 1.0 |  |
| Enjoying | 0.36 | 0.40 | 0.18 | 0.59 | 0.41 | 0.58 | 0.67 | 1.0 |
