# Supplementary material for: Key factors associated with oral health-related quality of life in Sri Lankan adolescents: a cross sectional study
Source: BMC Oral Health. 2021 Apr 29;21:218. doi: 10.1186/s12903-021-01569-1 (PMC8082852; doi:10.1186/s12903-021-01569-1)
Supplement: Supplementary file 4 — Additional file 4. Reliability analysis-corrected item total correlation. [file 12903_2021_1569_MOESM4_ESM.docx]

Supporting material 4

**Table 4** Reliability Analysis: Corrected item- total correlations (n= 220)

| **Items** | **Corrected item-total correlations** | **Cronbach’s alpha if item deleted** |
| --- | --- | --- |
| Impact on chewing and enjoying foods | 0.57 | 0.86 |
| Impact on talking and pronouncing clearly | 0.72 | 0.84 |
| Impact on cleaning teeth | 0.48 | 0.87 |
| Impact on good sleep without disturbances | 0.73 | 0.83 |
| Impact on being able to smile without embarrassment | 0.61 | 0.85 |
| Impact on maintaining usual emotional state without being irritable | 0.65 | 0.84 |
| Impact on school and household activities | 0.75 | 0.84 |
| Impact on enjoying time with friends | 0.59 | 0.85 |
