# Supplementary material for: Key factors associated with oral health-related quality of life in Sri Lankan adolescents: a cross sectional study
Source: BMC Oral Health. 2021 Apr 29;21:218. doi: 10.1186/s12903-021-01569-1 (PMC8082852; doi:10.1186/s12903-021-01569-1)
Supplement: Supplementary file 5 — Additional file 5. Concurrent validity test for the modified OIDP scores between different categories of related outcome variables. [file 12903_2021_1569_MOESM5_ESM.docx]

Supporting material 5

**Table 5** Concurrent validity test for the modified OIDP scores between different categories of related outcome variables (n= 220)

| **Variable** | **N** | **Mean** | **(SD)** | **P value^*^** |
| --- | --- | --- | --- | --- |
| Perceived oral treatment need |  |  |  |  |
| Yes | 82 | 2.60 | 3.85 | 0.003 |
| No | 103 | 1.56 | 4.00 |  |
| Don’t know | 35 | 2.08 | 3.91 |  |
| Perceived oral health problems |  |  |  |  |
| None | 145 | 0.96 | 1.45 | <0.001 |
| Little | 56 | 3.27 | 5.71 |  |
| Moderate | 15 | 5.41 | 5.91 |  |
| Severe | 4 | 10.96 | 2.45 |  |
| Very severe | 0 | - | - |  |

*Kruksal-Wallis test was performed
